# Chlamydia inhibits progesterone receptor mRNA expression in SHT-290 cells

**DOI:** 10.1530/RAF-20-0069

**Published:** 2021-03-09

**Authors:** Megan Brown, Mick Rae, Nick Wheelhouse

**Affiliations:** 1 School of Applied Sciences, Edinburgh Napier University, Sighthill Court, Edinburgh, Lothian, UK

**Keywords:** Chlamydia, stromal cell, decidualisation, progesterone

## Abstract

*Chlamydia trachomatis* is the most commonly diagnosed sexually transmitted infection in the UK, with over 200,000 positive diagnoses annually. The infection is thought to cause reproductive complications including problems in conceiving a pregnancy through to miscarriage and early or stillbirth. One potential reason Chlamydia may impact upon pregnancy is through disrupting the embryo implantation at the earliest stages of pregnancy is by altering the ability of specific cells that line the uterus called stromal cells to respond to the hormone progesterone, the hormone responsible for preparing the uterus for pregnancy. The results of this study showed that Chlamydial infection of these uterus lining stromal cells decreased the levels of specific progesterone sensitive markers which are associated with early embryo implantation, suggesting a loss of responsiveness to progesterone treatment. These changes were accompanied by a decrease in the levels of RNA for the progesterone receptor which is responsible for progesterone activity, suggesting that this is a potential mechanism through which Chlamydia could directly inhibit the effects of progesterone on uterine cells.

*Chlamydia trachomatis* (*Ct*) infection has significant long-term reproductive consequences, leading to a range of complications including infertility, miscarriage and preterm birth (Tang *et al.* 2020). While the mechanisms through which *Ct* leads to adverse pregnancy outcomes remain unknown, one potential cause is via disruption of uterine–embryo implantation. Successful implantation requires endometrial stromal cells (ESC) differentiation into secretory cells, a progesterone-driven process known as decidualisation. Chlamydial infection can reverse this process in primary ESCs (Giakoumelou *et al.* 2017). This study aimed to investigate potential mechanisms underpinning this process.

SHT290-cells (Telomerase-immortalised ESC line) were decidualised for 96 hours using a previously established protocol (Giakoumelou *et al.* 2017). Cells were exposed to *Ct* (serovar E) at a multiplicity of infection (MOI) 0.1, 1 or mock infection consisting of control media for 2 h before replacement with fresh decidualisation media (Giakoumelou *et al.* 2017). Chlamydial infection was quantified by qPCR, decidualisation was assessed by changes in cell morphology (for details see Supplementary file 1, see section on supplementary materials given at the end of the article) and by alterations in gene expression. 48h post-infection, RNA was isolated (RNeasy mini kit, Qiagen) as per manufacturer’s instructions. RNA samples were treated using DNAse 1 (Sigma Aldrich) before RT (Precision RT Premix, Primerdesign, UK). Quantitative real-time PCR was performed using 40ng of cDNA in a total volume of 20μl using Precision 2X qMasterMix with SYBR Green (PrimerDesign). Samples were analysed using 2−(ΔΔCt) quantification method relative to a mixed (pooled cDNA from each treatment) reference sample, using the geometric mean of stable housekeeping genes GAPDH and β-actin (Target and housekeeper primer sequences-Supplementary file 1).

*Ct* (MOI1) exposure was accompanied by a marked decrease in *Prolactin* mRNA (*P* < 0.05) and the endometrial receptivity marker *SPP-1* (*P* = 0.04), an essential implantation and receptivity mediator which is known to be expressed in primary ESC and during normal cycles within the endometrium (Marshall *et al.* 2011). Progesterone receptor mRNA expression was similarly reduced (*P* < 0.05) in *Ct* infected cells, conversely pro-inflammatory *COX-2* mRNA expression was robustly elevated (*P* =0.03) compared to non-infected cells. Testosterone is a likely key determinant of primary ESC decidualisation (Gibson *et al*. 2016) the effects of chlamydial infection on the expression of the steroid biosynthesis markers *CYP17A1* and *AKR1C3* was investigated. *CYP17A1* and *AKR1C3* were significantly downregulated by (*P* =0.02 and *P* =0.03, respectively) by MOI1 compared to the non-decidualised control (summarised in Fig. 1).

Findings of this study may suggest a possible pathway through which Ct may dysregulate endometrial decidualisation. Steroid hormones, particularly progesterone, are key drivers of ESC decidualisation. Progesterone removal results in a reversion of decidual phenotype characterised by changes in cellular morphology and reduced expression of prolactin, a key factor in the process of decidualisation (Evans & Salamonsen 2014), similar to the changes observed with *Ct* infection in the current study and in primary ESC (Giakoumelou *et al.* 2017). We suggest reduced progesterone receptor expression may dampen normal progesterone action in *Ct* infection. Intracrine biosynthesis of endometrial androgens during decidualisation has also been suggested to be a key driver of ESC decidualisation (Gibson *et al.* 2016). The results of the current study, demonstrating that infection reduces expression of androgen biosynthetic enzymes suggests that *Ct* also inhibits the ability of ESC to synthesise testosterone from progesterone. In addition to direct effects on steroid pathways, elevation in COX-2 expression may likely lead to an altered local prostanoid profile which may, in turn, promote a pro-inflammatory tone within uterine tissues.

**Figure 1:**
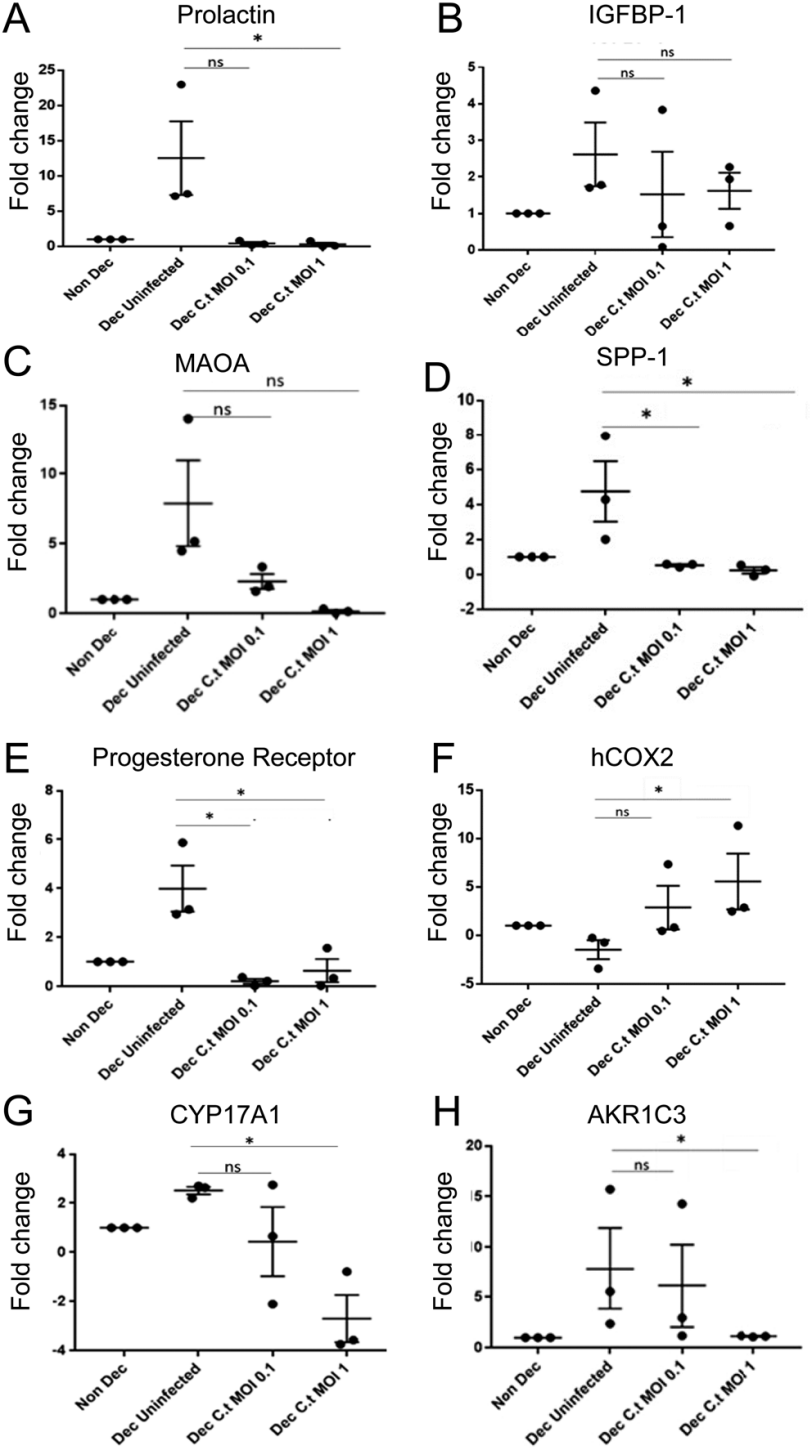
Changes in the expression of (A) Prolactin, (B) IGFBP-1, (C) MAOA, (D) SPP-1, (E) Progesterone receptor, (F) hCOX2, (G) CYP17A1, (H) AKR1C3 after exposure of decidualised (Dec) SHT-290 cells to medium alone (Uninfected) or Ct MOI 0.1, 1 for 48 h. Statistically-significant differences between control and infected cells are indicated by *P < 0.05. Data were analysed by one-way ANOVA. Comparisons between individual treatments were made using Tukey’s multiple comparison test.

This study has identified potential mechanisms through the progesterone pathway by which Ct may influence the responsiveness of ESCs to progesterone, negatively impacting upon implantation and successful pregnancy. Recognising that there may be other pathways through which Ct may impact implantation, more in-depth investigations are needed to determine the clinical significance of our findings.

## Supplementary Material

Supplementary Methods

Table S1 List of Primers

Table S2 Housekeepers Primer list

Figure S1. Chlamydial 16S in decidualised SHT-290 cells infected with C. trachomatis. Decidualisation was initialised using decidualised using 0.1mg/ml 8- Bromo-cAMP, 2% charcoal stripped FBS RPMI and 1 μM progesterone solution diluted in 100% ethanol. Cells were infected on day 4 of decidualisation and incubated for a further 48 hours, before lysis. p= 0.0006 (One-way Anova: Multiple Comparisons analysed using Tukeys test).

Figure S2. Immunostain fluorescence image of decidualised cells with and without infection with C. trachomatis serovar E. Cell nuceli staining blue using DAPI and beta-actin filaments stained in green using phalloidin. MOI=0.1.

## Declaration of interest

Nick Wheelhouse is an Associate Editor of Reproduction and Fertility. Nick Wheelhouse was not involved in the review or editorial process for this paper, on which he is listed as an author.

## Author contribution statement

M B performed the experiments and co-wrote the manuscript. M R helped with the statistical and intellectual interpretation of the data and provided critical revision of the manuscript. N W conceived and supervised the study and co-wrote the manuscript.

## References

[bib1] Evans J Salamonsen LA 2014 Decidualized human endometrial stromal cells are sensors of hormone withdrawal in the menstrual inflammatory cascade. Biology of Reproduction 90 14. (10.1095/biolreprod.113.108175) 24227758

[bib2] Giakoumelou S Wheelhouse N Brown J Wade J Simitsidellis I Gibson D Saunders PTK Horner P Entrican G Howie SEM 2017 Chlamydia trachomatis infection of human endometrial stromal cells induces defective decidualisation and chemokine release. Scientific Reports 7 2001. (10.1038/s41598-017-02223-z) 28515460 PMC5435679

[bib3] Gibson DA Simitsidellis I Cousins FL Critchley HO Saunders PT 2016 Intracrine androgens enhance decidualization and modulate expression of human endometrial receptivity genes. Scientific Reports 6 19970. (10.1038/srep19970) 26817618 PMC4730211

[bib4] Marshall E Lowrey J Macpherson S Maybin JA Collins F Critchley HO Saunders PT 2011 In silico analysis identifies a novel role for androgens in the regulation of human endometrial apoptosis. Journal of Clinical Endocrinology and Metabolism 96 E1746–E1755. (10.1210/jc.2011-0272) 21865353 PMC3380091

[bib5] Tang W Mao J Li KT Walker JS Chou R Fu R Chen W Darville T Klausner J Tucker JD 2020 Pregnancy and fertility-related adverse outcomes associated with Chlamydia trachomatis infection: a global systematic review and meta-analysis. Sexually Transmitted Infections 96 322–329. (10.1136/sextrans-2019-053999) 31836678 PMC7292777

