# Supplementary material for: Chlamydia inhibits progesterone receptor mRNA expression in SHT-290 cells
Source: Reprod Fertil. 2021 Mar 9;2(1):L9–L11. doi: 10.1530/RAF-20-0069 (PMC8812455; doi:10.1530/RAF-20-0069)
Supplement: Supplementary Methods [file supplementary_material.pdf]

Supplementary Methods

Infection of SHT-290 Cell with *C. trachomatis* Serovar E

Frozen aliquots of *C. trachomatis* serovar E stocks were thawed at room temperature. A measured volume of 20 µl of *C. trachomatis* stock was added to 20ml of pre-warmed decidualising/non-decidualising media), for a *C. trachomatis* concentration suitable to obtain an Multiplicity of infection (MOI) of 1. 2 ml of this *C. trachomatis* infected media was diluted in a total volume of 20 ml of phenol free RPMI-1640, supplemented as above, in order to obtain an MOI of 0.1. All infections were carried out on day 4 after the initiation of decidualisation. For the infection the medium was removed from wells and replaced with media containing *C. trachomatis* serovar E at either an MOI or 1 or 0.1. Cells were left to incubate at 37°C for 2 hours at a CO_2 concentration of 5%. The media was then removed from the wells and replaced with fresh medium. Cells were then incubated at 37°C/5% CO_2 for a further 48 hours.

qPCR of C. trachomatis infected SHT-290 cells

Prior to quantification of chlamydial gene copy number, total genomic DNA was isolated using the DNeasy Blood and Tissue kit as per manufacturer's instructions (Qiagen). Genome copy number was quantified using 16S rDNA qPCR (Lienard et al., 2011). Each reaction had a total volume of 22 µl per well containing 9.6 µl of ROX mastermix (PrimerDesign LTD), 10 µl of nuclease-free water, 0.2 µl of 16S Chlamydial forward and reverse primers, 0.2 µl of Chlamydial 16S Probe and 2 µl of sample DNA/ Standard. The plate was centrifuged at 500 g for 5 seconds to remove any air bubbles. Samples were analysed in a qPCR thermocycler (Applied Biosystems) with an initial holding stage of 3 minutes at 95°C, followed by 40 cycles of 15 seconds at 95°C, 15 seconds at 67°C and 15 seconds at 72°C. Genomic copy numbers were quantified against a standard curve generated from a recombinant plasmid (Wheelhouse et al., 2014).

Fluorescent Microscopy/Confocal Imaging of Decidualised SHT-290 cells

Prior to staining, cells were seeded in 8 well Chamber slides (Thermo Fisher Scientific Ltd (FalconTM)). SHT-290 cells were plated onto chamber slides, using protocol from section 2.4 at a concentration of 8x104 cells per well in 0.5 ml of medium. After decidualisation, the supernatants were removed, and each well washed using sterile PBS. The chamber slide was fixed in acetone for five minutes at room temperature and stored at -20oC for 20 minutes. The slide was washed again using PBS to remove any excess solutions, the gasket of the chamber slide was removed, and slide was submerged in 2% (w/v) BSA

solution was used to block non-specific binding, and incubated in the dark at room temperature for 1 hour. The slide was then washed in PBS solution. A volume of 0.75 µl of Alexa Fluor 488 Phalloidin stain (Invitrogen, UK) was diluted in 1.5 ml of PBS and mixed by flicking. The phalloidin solution was added to the slide to stain the actin and incubated at room temperature in the dark for 20 minutes. Slide was washed again three times using PBS and the slide was covered using Antifade Mounting Media with DAPI (Vectashield, Peterborough, UK) to stain the cell nuclei. A glass cover slip was placed on top of the chamber slide. The slide was dried and sealed using nail polish. The slides were analysed and images were captured using an LSM 880 confocal microscope (Carl Zeiss Ltd, UK), using the identical laser/lighting settings across all samples. Zen black software (Carl Zeiss Ltd) was used to process the images.

Gene Expression real-time PCR conditions

The samples were analysed in a Step One Plus qPCR thermal cycler (Applied BiosystemsTM) with an initial holding step of a minute at 90°C and 40 cycles of 15 seconds at 60°C followed by 15 seconds at 95°C and final stage of 5 minutes at 95°C.

References

LIENARD, J., CROXATTO, A., AEBY, S., JATON, K., POSFAY-BARBE, K., GERVAIX, A. & GREUB, G. 2011. Development of a new chlamydiales-specific real-time PCR and its application to respiratory clinical samples. *J Clin Microbiol,* 49**,** 2637-42.

WHEELHOUSE, N., COYLE, C., BARLOW, P. G., MITCHELL, S., GREUB, G., BASZLER, T., RAE, M. T. & LONGBOTTOM, D. 2014. Waddlia chondrophila infects and multiplies in ovine trophoblast cells stimulating an inflammatory immune response. *PLoS One,* 9**,** e102386.
