# Supplementary material for: Chlamydia inhibits progesterone receptor mRNA expression in SHT-290 cells
Source: Reprod Fertil. 2021 Mar 9;2(1):L9–L11. doi: 10.1530/RAF-20-0069 (PMC8812455; doi:10.1530/RAF-20-0069)
Supplement: Table S1 List of Primers [file supplementary_table_1.pdf]

Table S1 List of Primers

Primer name	Primer Sequence (5' – 3')	Primer Length	GC content	T_m (°C)
Prolactin-Fwd[a]	AAAGGATCGCCATGGAAAG	19	68.4%	63.1
Prolactin-Rv[a]	GCACAGGAGCAGGTTTGAC	21	52.4%	59.8
IGFBP-1 Fwd	AGGCTCTCCATGTCACCA	18	55.5%	58.9
IGFBP1 Rv	CTCCTGATGTCTCCTGTGCCTT	22	54.%	62.1
SPP-1 Fwd[a]	GAGGGCTTGGTTGTCAGC	18	61.1%	57.7
SPP-1 Rv[a]	CAATTCTCATGGTAGTGAGTTTTCC	25	40.0%	58.9
MAOA Fwd[a]	GGCCACATGTTCGACGTAGT	20	55.0%	59.4
MAOA Rv[a]	CATGCCAATCTCTTCTCTTGG	21	47.6%	58.3
EDNRB Fwd[a]	ATCGTCATTGACATCCCTATCA	22	45.4%	57.8
EDNRB Rv[a]	GCTTACACATCTCAGCTCCAAA	22	45.4%	58.1
AKR1C3 Fwd[a]	TGGGTTCCGCCATATAGATT	20	45.0%	57.8
AKR1C3 Rv[a]	TCGATGAAAAGTGGACCAAA	20	40.0%	57.5
CYP17A1 Fwd[a]	TGGCTTTCCTGGTGCACAATC	21	52.3%	64.0
CYP17A1 Rv[a]	TGAAAGTTGGTGTTCGGCTGAAG	23	47.8%	64.2
16S Fwd	GCTGACGGCGTGGATGAGGC	20	65%	61.3
16S Rv	CCTACGCGCCCTTTACGCCC	20	70%	68.4
Progesterone Receptor Fwd	CGCGCTCTACCCTGCACTC	19	68.4%	63.0
Progesterone Receptor Rv	TGAATCCGGCCTCAGGTAGTT	21	52.3%	61.7
hCOX2 Fwd	GAATGGGGTGATGAGCAGTT	20	50.0%	58.4
hCOX2 Rv	CAGAAGGGCAGGATACAGC	19	52.6%	56.8

a Primers (Gibson et al., 2016)
