# Supplementary material for: Chlamydia inhibits progesterone receptor mRNA expression in SHT-290 cells
Source: Reprod Fertil. 2021 Mar 9;2(1):L9–L11. doi: 10.1530/RAF-20-0069 (PMC8812455; doi:10.1530/RAF-20-0069)
Supplement: Table S2 Housekeepers Primer list [file supplementary_table_2.pdf]

Table S2 Housekeepers Primer list

Primer name	Primer Sequence (5' – 3')	Primer Length	GC content	T_m (°C)
GAPDH Fwd	AAGCTCATTTCCTGGTATGACA	22	45.4%	57.2
GAPDH Rv	TCTTACTCCTTGGAGGCCATGT	22	50.0%	60.8
β-Actin Fwd	GGACTTCGAGCAAGAGATGG	20	55.0%	58.4
B-Actin Rv	AGGAAGGAAGGCTGGAAGAG	20	55.0%	58.4
