# Supplementary material for: Chlamydia inhibits progesterone receptor mRNA expression in SHT-290 cells
Source: Reprod Fertil. 2021 Mar 9;2(1):L9–L11. doi: 10.1530/RAF-20-0069 (PMC8812455; doi:10.1530/RAF-20-0069)
Supplement: Figure S1. Chlamydial 16S in decidualised SHT-290 cells infected with C. trachomatis. Decidualisation was initialised using decidualised using 0.1mg/ml 8- Bromo-cAMP, 2% charcoal stripped FBS RPMI and 1 μM progesterone solution diluted in 100% ethanol. Cells were infected on day 4 of decidualisation [file supplementary_figure_1.pdf]

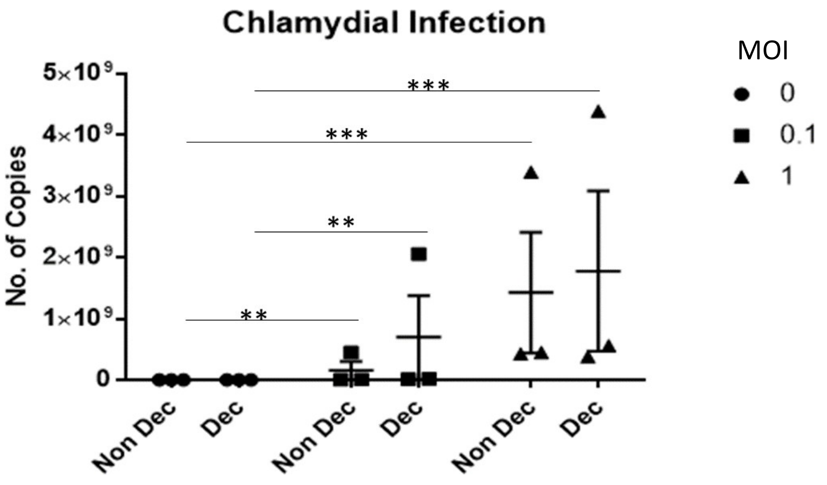

Figure S1. Chlamydial 16S in decidualised SHT-290 cells infected with C. trachomatis. Decidualisation was initialised using decidualised using 0.1mg/ml 8- Bromo-cAMP, 2% charcoal stripped FBS RPMI and 1 μM progesterone solution diluted in 100% ethanol. Cells were infected on day 4 of decidualisation and incubated for a further 48 hours, before lysis. p= 0.0006 (One-way Anova: Multiple Comparisons analysed using Tukeys test).
