# Supplementary material for: Chlamydia inhibits progesterone receptor mRNA expression in SHT-290 cells
Source: Reprod Fertil. 2021 Mar 9;2(1):L9–L11. doi: 10.1530/RAF-20-0069 (PMC8812455; doi:10.1530/RAF-20-0069)
Supplement: Figure S2. Immunostain fluorescence image of decidualised cells with and without infection with C. trachomatis serovar E. Cell nuceli staining blue using DAPI and beta-actin filaments stained in green using phalloidin. MOI=0.1. [file supplementary_figure_2.pdf]

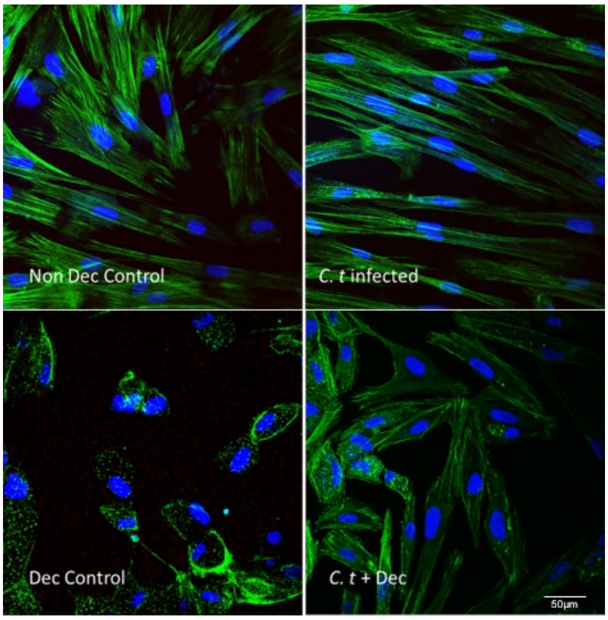

Figure S2. Immunostain fluorescence image of decidualised cells with and without infection with *C. trachomatis* serovar E. Cell nuceli staining blue using DAPI and beta-actin filaments stained in green using phalloidin. MOI=0.1.
